# Supplementary material for: Open-Bundle Structure as the Unfolding Intermediate of Cytochrome c′ Revealed by Small Angle Neutron Scattering
Source: Biomolecules. 2022 Jan 7;12(1):95. doi: 10.3390/biom12010095 (PMC8774185; doi:10.3390/biom12010095)
Supplement: Supplementary file 1 [file biomolecules-12-00095-s001.zip › biomolecules-1460435-supplementary.pdf]

Supplementary Material

Determination of the Open-Bundle Structure of Cytochrome *c*' as a Folding/Unfolding Intermediate by Small-Angle Neutron Scattering

Takahide Yamaguchi,[1,2] Kouhei Akao,[1] Alexandros Koutsioubas,[3] Henrich Frielinghaus,[3] and Takamitsu Kohzuma*[1,2]

[1]Institute of Quantum Beam Science, Graduate School of Science and Engineering, Ibaraki University, 2-1-1 Bunkyo, Mito, Ibaraki 310-8512, Japan

[2]Frontier Research Center of Applied Atomic Sciences, Ibaraki University, 162-1 Shirakata, Tokai, Ibaraki 319-1106, Japan

[3]Jülich Centre for Neutron Science at Heinz Maier-Leibnitz Zentrum, Forschungszentrum Jülich GmbH, Lichtenbergstrasse 1, Garching D-85747, German

Table of Contents

Figure S1. SDS-PAGE of purified Cyt *c*' on a 12.5% gel……………….……… page 3

Figure S2. Electronic absorption spectra of Cyt *c*' at pD 1.7, 6.4, 9.6, and ~13. The spectral patterns are annotated by charge-transfer 3 (CT3), Soret, CT2, Q, and CT 1 at short wavelengths. The samples were prepared by diluting the samples following SANS experiments……………………………………………………………………… page 3

Figure S3. Curves obtained by *ab initio* analyses (red lines) and data points used for each condition (squares: pD 6.4, circles: pD 9.6, triangles: pD ~13)…...………… page 4

Figure S4. The estimation of I(0) values by extrapolation of the I(*Q*) SANS curves (top panels) and Guinier plots (bottom panels) at pD 1.7 (A, red), 6.4 (B, orange), 9.6 (C, green), and ~13 (D, blue). The black solid lines in the top panels are fitted I(Q) SANS curves by Fourier transformation of *P*(*r*) at each pD value, and the black dashed lines in the bottom panels were obtained from linear Guinier fitting. The I(0) point at each pD value is marked by a diamond on the vertical axis.…...……………………… page 4

Figure S5. Fitting of BUNCH simulations (red line) versus the experimental SANS curve at pD ~13 (blue circle)……………………………...……………………… page 5
Table S1. Details of sample used in SANS experiment…...…………………… page 6
Table S2. SANS data collection parameters……..…………………………. page 6
Table S3. Software employed for SANS data reuduction, analysis, and interpretation…..……………………………………………………………. page 7
Table S4. Structural parameters determined from SANS curves…..………. page 7

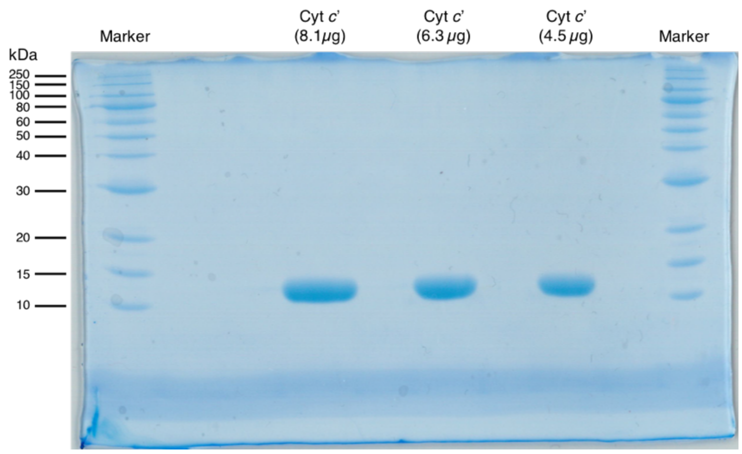

Figure S1. SDS-PAGE of purified Cyt *c*' on a 12.5% gel.

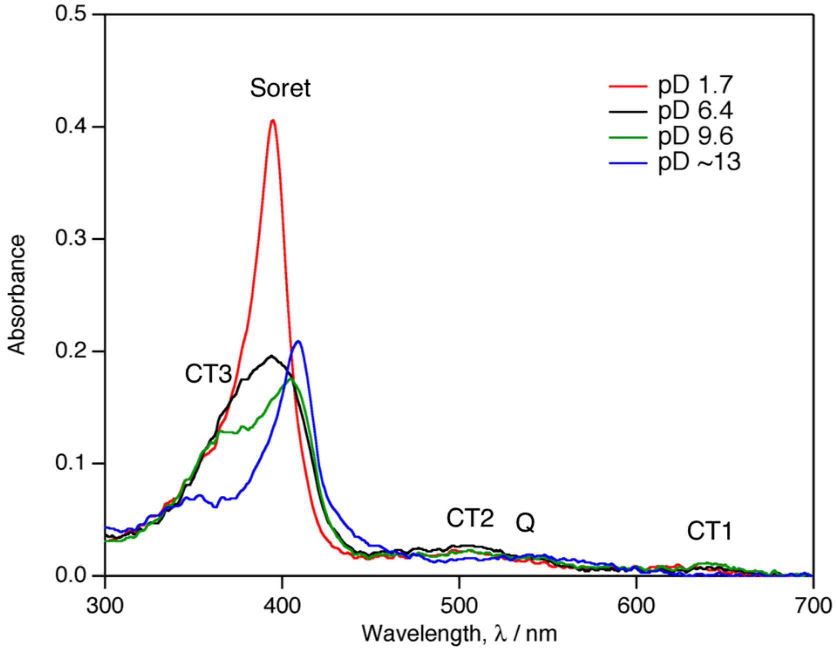

Figure S2. Electronic absorption spectra of Cyt c' at pD 1.7, 6.4, 9.6, and ~13. The spectral patterns are annotated by charge-transfer 3 (CT3), Soret, CT2, Q and CT 1 at short wavelengths. The samples were prepared by diluting the samples following SANS experiments.

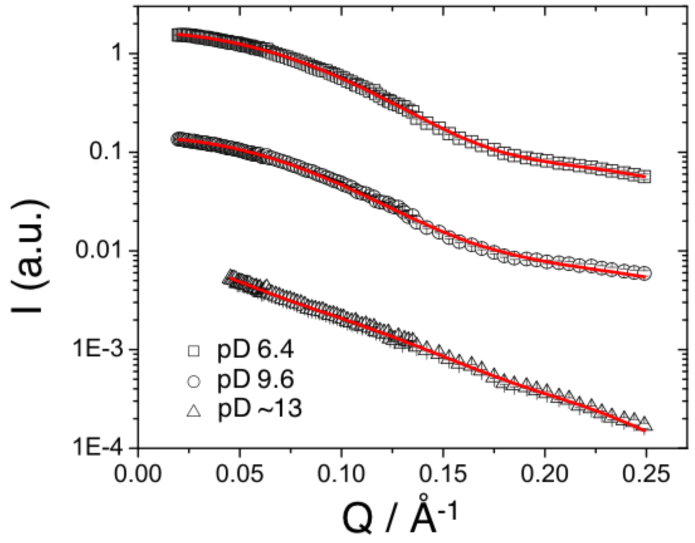

Figure S3. Curves obtained by ab initio analyses (red lines) and data points used for each condition (squares: pD 6.4, circles: pD 9.6, triangles: pD ~13).

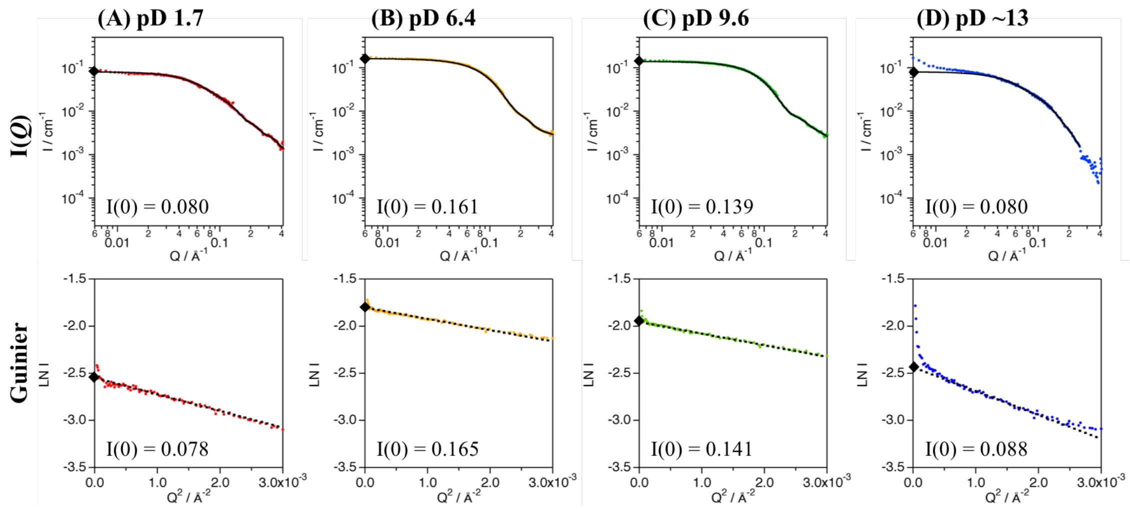

Figure S4. The estimation of I(0) values by extrapolation of the I(Q) SANS curves (top panels) and Guinier plots (bottom panels) at pD 1.7 (A, red), 6.4 (B, orange), 9.6 (C, green), and ~13 (D, blue). The black solid lines in the top panels are fitted I(Q) SANS curves by Fourier transformation of $P(r)$ at each pD value, and the black dashed lines in the bottom panels were obtained from linear Guinier fitting. The I(0) point at each pD value is marked by a diamond on the vertical axis.

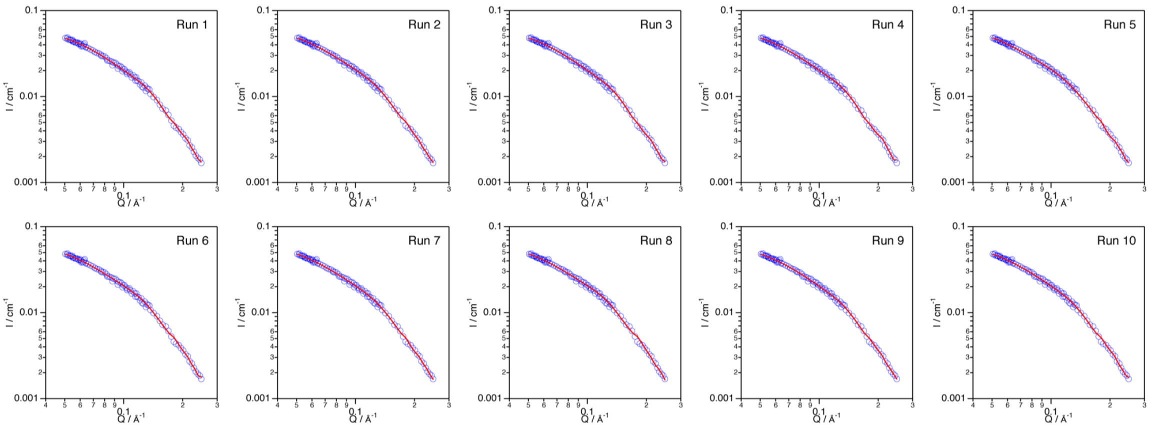

Figure S5. Fitting of BUNCH simulations (red line) versus the experimental SANS curve at pD ~13 (blue circle).

Table S1. Details of sample used in SANS experiment.

	Cytochrome *c′*
Organism	*Alcaligenes xylosoxidans*
Source	NCIMB 11015
UniProt Sequence ID (residues in construct)	P00138(1-127)
Molar Extinction Coefficient / M^{-1} cm^{-1}	96,000 (Soret band)
Molecular Weight (Da) for Monomer	14227.2 (determined by ESI-MS)
Concentration / mg mL^{-1}	5.3-5.5

Table S2. SANS data collection parameters.

Instrument	KWS-1 (JCNS/FRM-II)
Wavelength / Å	5 Å with a spread $\Delta\lambda/\lambda$ = 10%
Beam size / µm	10,000 x 10,000
Camera length	C+D = 8+8 m (Low-Q), 4+4 m (Medium-Q, transm.), 4+1.5 m (High-Q)
Q measurement range / $Å^{-1}$	0.005997535 - 0.4162468
Absolute scaling method	Comparison with scattering intensity of secondary standard Plexiglas
Normalization	Secondary Plexiglas
Exposure time	4800 s (8m), 900 s (4m), 7200 s (1.5m)
Sample temperature / K	293 K

Table S3. Software employed for SANS data reduction, analysis, and interpretation.

SANS data reduction	qtiKWS
Basic analyses: Guinier, P(r)	PRIMUS in ATSAS v. 2.8.2
Shape/bead modelling	DENFERT v.2
Preparation of volumetric map/model docking	pdb2vol/collage in Situs v.2.8. package
Numerical model fitting	IGOR Pro v. 6.04, Maple

Table S4. Structural parameters determined from SANS curves.

	pD 1.7	pD 6.4	pD 9.6	pD ~13
Guinier analysis				
$I(0)$ / cm^{-1}	0.078±0.001	0.165±0.001	0.141±0.001	0.088±0.002
R_g / Å	23.02±0.57	18.93±0.45	19.24±0.49	27.58±0.59
QR_g limit	0.73-1.03	0.60-0.84	0.61-0.86	0.87-1.23
P(r) analysis				
$I(0)$ / cm^{-1}	0.0804±0.0004	0.1609±0.0008	0.1389±0.0002	0.0804±0.0014
R_g / Å	25.03±0.19	18.24±0.08	18.10±0.05	25.54±0.60
d_{max} / Å	86.21	54.87	53.16	89.52
Q range / $Å^{-1}$	0.0197-0.4160	0.0197- 0.4160	0.0197-0.4160	0.0321-0.2540
